# Supplementary material for: Content-rich biological network constructed by mining PubMed abstracts
Source: BMC Bioinformatics. 2004 Oct 8;5:147. doi: 10.1186/1471-2105-5-147 (PMC528731; doi:10.1186/1471-2105-5-147)
Supplement: Additional File 2 — The original results of the above study (non-essential files are deleted to keep the file size under the limit set by BMC bioinformatics). [file 1471-2105-5-147-S2.bz2 › chilibotAdditionalFile2/dip05/357ID9062193E4999/html/IL1R1_IL1B.html]

 


 **IL1R1** and **IL1B** 
  
Found 453 abstracts in PubMed, retrieved 05.  
 

 What does Google say? 
 PDF only 
| .edu only 

---

- J Neurosci Res, 2003   **Lymphocyte infiltration in the injured brain Role of proinflammatory cytokines.** .
  Studies using mouse axotomised facial motoneuron model show a strong and highly selective entry of CD3 lymphocytes into the affected nucleus, with a maximum at Day 14, which coincides with the peak of neuronal cell death, microglial phagocytosis, and increased synthesis of interleukin 1 beta **[IL1B]** IL1beta , tumour necrosis factor alpha TNFalpha and interferon gamma IFNgamma .
  We explored the possible involvement of these cytokines during the main phase of lymphocyte recruitment into the axotomised facial motor nucleus 7 21 days after nerve cut using mice homozygously deficient for IL1 receptor type 1 **IL1R1** , TNF receptor type 1 TNFR1 , type 2 TNFR2 and type 1 and 2 TNFR1&2 , IFNgamma receptor type 1 IFNgammaR1 , and the appropriate controls for the genetic background.
  Transgenic deletion of **IL1R1** led to a 54% decrease.
  of TNFR2 to a 44% reduction in the number of CD3 T cells in the axotomised facial motor nucleus, with a similar relative decrease at Day 4, and 21.
  Deletion of TNFR1 or IFNgammaR1 had no significant effect.
  Deletion of both TNFR1 and 2 TNFR1&2 caused a somewhat stronger, 63% decrease than did TNFR2 deletion alone, but this could be due to an almost complete inhibition of neuronal cell death.
  No mutations seemed to inhibit aggregation of CD3 T cells around glial nodules consisting of Ca ion binding adaptor protein 1 IBA1 phagocytotic microglia and neuronal debris.
  Altogether, the current data show the importance of **IL1R1** and TNFR2 as the key players during the main phase of lymphocyte recruitment to the damaged part of the central nervous system.

  - Br J Pharmacol, 2003   **Effect of a kinin B 2 receptor antagonist on LPS and cytokine induced neutrophil migration in rats.** .
    1 This study examines the involvement of kinins in neutrophil migration into rat subcutaneous air pouches triggered by lipopolysaccharide LPS , as well as the putative roles played by kinin B 1 and B 2 receptors, tumour necrosis factor alpha TNF alpha , interleukin 1 beta **[IL1B]** IL 1beta and selectins in this response.
    2 LPS 5 ng to 10 micro g cavity 1 injected into the 6 day old pouch induced a dose and time dependent neutrophil migration which peaked between 4 and 6 h, and was maximal following the dose of 100 ng cavity 1 saline 0.46 0.1.
    LPS 43 3.70 x 10 6 cells cavity 1 at 6 h .
    3 Bradykinin BK 600 nmol injected into the pouch of saline treated rats induced only modest neutrophil migration 0.73 0.16 x 10 6 cells cavity 1 .
    A more robust response to BK 3.2 0.25 x 10 6 cells cavity 1 was seen in animals pretreated with captopril, but this was still smaller than the responses to IL 1beta or TNF alpha 15 pmol 23 2.2 x 10 6 and 75 pmol 29.5 2 x 10 6 cells cavity 1 , respectively .
    Nevertheless, the B 1 agonist des Arg 9 BK 600 nmol failed to induce neutrophil migration.
    4 HOE 140 1 and 2 mg kg 1 , a B 2 receptor antagonist, reduced LPS induced neutrophil migration.
    HOE 140 also reduced the neutrophil migration induced by BK, but had no effect on the migration promoted by IL 1beta or TNF alpha.
    des Arg 9 Leu 8 BK, B 1 receptor antagonist was ineffective in changing neutrophil migration caused by any of these stimuli.
    5 Neutrophil migration induced by LPS or BK was reduced by interleukin 1 receptor **[IL1R1]** antagonist IL 1ra 1 mg kg 1 , sheep anti rat TNF serum anti TNF serum 0.3 ml cavity 1 , and the nonspecific selectin inhibitor fucoidin 10 mg kg 1 .
    6 TNF alpha levels in the pouch fluid were increased by LPS or BK injection, peaking at 0.5 1 h and gradually declining thereafter up to 6 h.
    IL 1beta levels increased steadily throughout the 6 h period.
    HOE 140 markedly inhibited the rise in IL 1beta and TNF alpha levels in pouch fluid triggered by both stimuli.
    7 These results indicate that BK participates importantly in selectin dependent neutrophil migration into the air pouch triggered by LPS in the rat, by stimulating B 2 receptors coupled to synthesis release of TNF alpha and IL 1beta.
    British Journal of Pharmacology 2003 13 71 278.
    doi 10.1038 sj.bjp.0705236

    - Endocrinology, 2003   **Interleukin 6 is an afferent signal to the hypothalamo pituitary adrenal axis during local inflammation in mice.** .
      The cytokines IL 1 and IL 6 are able to induce prostaglandin PG dependent activation of the hypothalamo pituitary adrenal axis HPAA and are thought to play key roles in immune neuroendocrine interactions during inflammation.
      The present study shows that inflammation induced by im injection of turpentine TPS in the hind limb of mice causes an increase in the plasma concentration of IL 6, but not that of IL 1 alpha or IL 1 beta **[IL1B]**, together with a prolonged >18 h activation of the HPAA.
      IL 6 plays a causal role in the TPS induced elevation in HPAA activity, because the sustained 8 18 h increases in 1 plasma corticosterone, 2 plasma ACTH, and 3 induction of c Fos in the hypothalamic paraventricular nucleus are all markedly blunted in IL 6 deficient IL 6 mice.
      Peripheral administration of a neutralizing IL 6 antiserum inhibited the plasma corticosterone response of normal C57BL 6 mice to hind limb inflammation to an extent similar to that seen in IL 6 mice, suggesting that the IL 6 responsible for the increased HPAA activity is produced, or acts, on the blood side of the blood brain barrier.
      We also show that IL 6 in the circulation is induced almost exclusively at the local inflammatory site, where IL 1 beta **[IL1B]** is produced.
      Induction of IL 6 and activation of the HPAA are dependent upon prior activation of an IL 1 type I receptor **[IL1R1]**, as both are inhibited in type I IL 1 receptor deficient mice.
      Furthermore, hind limb inflammation induced cyclooxygenase 2 protein expression around the cerebrovasculature of normal IL 6 , but not IL 6 , mice.
      Based on these data, we propose that IL 6 is produced at the local inflammatory site under the control of IL 1 beta **[IL1B]** and is the circulating afferent signal that is in part responsible for elevated HPAA activity, possibly acting via eicosanoid production within the cerebrovasculature.
